# Coexistence of Axial Spondyloarthritis and Idiopathic Inflammatory Myopathy

**DOI:** 10.1155/2020/8840642

**Published:** 2020-10-07

**Authors:** Yongpeng Ge, Linrong He

**Affiliations:** Department of Rheumatology, China-Japan Friendship Hospital Yinghua East Road, Chaoyang District 100029, Beijing, China

## Abstract

Both axial spondyloarthritis (axSpA) and idiopathic inflammatory myopathy (IIM) are infrequent, and their coexistence is even rarer; there are a few reported cases in the literature. The aim of this study was to assess their association and clinical and laboratory features in our patients. The clinical data of patients with axSpA and IIM diagnosed in China-Japan Friendship Hospital from July 2015 to February 2019 were retrospectively analyzed. This study included 7 patients with axSpA who met the IIM criteria, including 3 males and 4 females. The age of onset was 16 to 39 years. Four patients were HLA-B27 positive, and three were negative. All patients were first diagnosed as axSpA, and then IIM was detected after 0.5–20 years (mean ± SD, 9.9 ± 5.0 years). After being diagnosed to have axSpA and IIM, those patients were given prednisone and immunosuppressant drugs, and their symptoms gradually improved. Our study provides further evidence of the coexistence of IIM with axSpA. In patients with axSpA who have skin rash, interstitial lung disease (ILD), myalgia, or muscle weakness, we should suspect that they may have IIM.

## 1. Introduction

Axial spondyloarthritis (axSpA) is a chronic inflammatory disease of the axial skeleton manifested by inflammatory back pain and progressive stiffness of the spine. Idiopathic inflammatory myositis (IIM) is a chronic inflammatory disease of unknown etiology that may affect the skin, muscle, lungs, and/or other organs of the body. These two rheumatologic diseases, which have a different aetiopathogenesis as well as diverse clinical and genetic characteristics, are rarely seen together. To the best of our knowledge, there are a few reported cases of the coexistence of IIM and axSpA in the literature studies [1–3]. Here, we report several cases where these two diseases coexist. We also intend to review the clinical and laboratory features of previously reported cases.

## 2. Patients and Methods

Patients with IIM combined with axSpA were admitted to the rheumatology department of China-Japan Friendship Hospital from July 2015 to February 2019. This study was approved by the Medical Ethics Committee of China-Japan Friendship Hospital. We used the new axSpA classification standard published by the SpondyloArthritis International Society (ASAS) (2009) [4, 5]. The diagnosis of IIM met the Bohan and Peter classification criteria of 1975 [6, 7] or 2004 ENMC [8].

Laboratory tests included white blood cell (WBC), neutrophil, hemoglobin (HGB), blood platelet (PLT), erythrocyte sedimentation rate (ESR), C-reactive protein (CRP), HLA-B27, alanine transaminase (ALT), aspartate aminotransferase (AST), lactate dehydrogenase (LDH), serum creatine kinase (CK), serum ferritin, and complement level. Myositis-specific antibodies (MSAs) were detected by western blotting in all patients. All patients had been tested for antinuclear antibody (ANA), double-stranded DNA (ds-DNA), rheumatoid factor (RF), antineutrophil cytoplasmic antibody (ANCA), and anticardiolipin (ACL) antibodies to exclude connective tissue disease (CTD) and other diseases. Echocardiographies, cardiac color Doppler ultrasound, sacroiliac joint CT and/or MRI, and muscle biopsy were also performed. The grading system of sacroiliitis by MRI and CT was based on the 1984 modified New York criteria; Grade 1, suspicious changes; Grade 2, minimal abnormality, small localized areas with erosion or sclerosis, without alteration in the joint width; Grade 3, unequivocal abnormality, moderate or advanced sacroiliitis with one or more erosions, evidence of sclerosis, widening, narrowing, or partial ankylosis; Grade 4, severe abnormality, total ankylosis.

### 2.1. Statistical Methods

Descriptive methods were used for statistical analysis.

## 3. Results

### 3.1. General Clinical Data

From July 2015 to February 2019, a total of seven patients were included in our study according to the axSpA and IIM criteria. Among the 7 patients with IIM complicated with axSpA, there were 4 females and 3 males, onset age of axSpA from 16 to 39 years old, and onset age of IIM onset from 26 to 55 years old. All 7 patients were diagnosed as axSpA when the symptoms of spinal and peripheral joints firstly occurred, and then were found IIM positive years later (0.5–20 years; mean ± SD, 9.9 ± 5.0 years). Among them, no cases were complicated with tumor or other rheumatic disease. Those patient data are shown in Tables 1 and 2.

### 3.2. Clinical Features of axSpA

All of them had inflammatory low-back pain, of which 3 patients (42.9%) had peripheral joint involvement. Three (42.9%) had family history of axSpA, and four (57.1%) carried HLA-B27. CT and/or MRI showed that the grade of sacroiliac joint lesions were 2–4. Three patients (42.9%) suffered with iritis. One patient had received recombinant soluble TNF-*α* receptor fusion protein (etanercept) and sulfasalazine (SSZ) for two years, and one patient received treatment with infliximab (IFX) intermittently 5 years ago. Other patients did not receive regular treatment.

### 3.3. Clinical and Laboratory Characteristics of IIM

Three of them (42.9%) had typical skin rash, such as symptoms of heliotrope sign, Gottron's rash. In the IIM patients, five (71.4%) showed clinical symptoms of muscular weakness, and two (28.6%) had slightly muscle weakness. Elevated CK occurred in four patients. Two carried anti-MDA5 antibody, one carried SRP antibody, and one carried PL-7 antibody. Electromyography (EMG) showed myogenic changes in four patients (57.1%), and inflammatory exudation was found in three patients (42.9%) by muscle MRI. Muscle biopsy was performed in all patients, three of which showed obviously necrotizing muscle fiber and three perifascicular atrophy in their muscular pathology. Three patients (42.9%) complicated with interstitial lung disease (ILD) diagnosed by high-resolution CT (HRCT), two of which had dyspnea. According to the criteria of the 2004 ENMC, three (42.9%) were immune-mediated necrotizing myositis (IMNM) without statins exposure, three (42.9%) were dermatomyositis (DM) including one clinically amyopathic dermatomyositis (CADM), and one (14.2%) belonged to antisynthetase syndrome (ASS). Six patients received glucocorticoid and immunosuppressant agents, such as tacrolimus (TAC), methotrexate (MTX), and cyclosporine (CsA), and one patient was given glucocorticoid only when the IIM was diagnosed. These patients received long-term monitoring. The follow-up time ranged from 12 to 54 months (median 36 months). All patients survived and responded well to glucocorticoid and/or immunosuppressive agents and experienced improvement in skin rash and/or myositis. HRCT of the 3 patients with ILD improved significantly. No malignant tumor was found during this long-term follow-up period.

## 4. Discussion

AxSpA is a chronic inflammatory disease of the type of axial spondyloarthropathy, which mainly involves joint axis, sacroiliac joint with different degrees of involvement of peripheral joints, tendons, ligaments, articular cartilage, and ligament attachment points, causing fibrous and bony rigidity. The etiology is unknown, and it is related to genetic, environmental factors, including the HLA-B27, which is closely related to the disease occurrence and has a clear tendency of familial aggregation. IIM is an idiopathic inflammatory myopathy with the common characteristics of proximal skeletal muscle weakness, diverse skin rash, and muscle inflammation. The exact pathogenesis remains unclear, which may be related to infection, genetic factors, and immune abnormalities. However, all cases of axSpA and diffuse CTD merger were reported, and only 3 cases of SpA and IIM merger were reported abroad [1–3]. In 1983, Prohaska and Moritz reported an ankylosing spondylitis (AS) in a 58-year-old male patient with definite clinical symptoms, imaging changes, and positive HLA-B27, followed by a combination of DM and atypical gout [1]. In addition, Chadrasekhara et al. reported one patient with SLE, DM, and AS [2]. In 1988, Sattar et al. reported a case with RA, AS, and DM, and HLA was classified as HLA-A2, A9, B8, B27, DR3, and DR antigens, suggesting that the incidence of the disease may be related to genetic susceptibility [3]. Unfortunately, in this study, we did not test other HLA antigens other than B27.

Here, we report seven cases of patients affected by axSpA associated with IIM in our hospital, which are the first report from China and the report with the largest number of patients so far. This was not similar to other axSpA patients alone, and most of the seven axSpA we have with IIM patients were also young women, which is different from male-dominated axSpA. Most of the patients had long-lasting diseases and therefore discontinued treatment. In our report, three-seventh patients were HLA-B27 negatives, unlike axSpA alone which was HLA-B27 predominance. We speculate that inflammatory process may play a crucial role in it. Hopkins GO et al. reported muscle biopsy was carried out in 20 patients with classical AS, and they found that all biopsies have varying degrees of change, 14 out of 16 patients had weakness of quadriceps strength, and half of those showed lower mean power frequency in EMG, but only two patients had raised CK [9]. Faus-Riera S et al. reported that the plasma CK of AS patient was higher than that of the controls. Myopathic EMG pattern was found in 46.4% AS patients, and nonspecific histological changes were found in 66% patients [10]. They speculated that muscles were further affected as a consequence of pain suppression and reduced activity [10].

In our report, axSpA appeared in combination with different types of IIM. Although biologics can cause IIM, two patients had been off biologics for two years before IIM. Therefore, we speculate that biological agents may not be involved in the pathogenesis of IIM.

Regarding the causes of CTD and SpA in the same body and whether there is a common basis for the disease, the current research has not yet reached a clear conclusion. Most studies speculated that environmental factors played an important role in the genetic susceptibility, and the body immune dysfunction may lead to the disease. In this case, the combination of the two diseases, along with the development of the disease, whether it will be combined with other autoimmune diseases, requires close follow-up. However, myositis may be caused by other causes than the autoimmune disease. Recently, one study investigated the risk of immune-mediated inflammatory diseases in patients with AS, but the results showed that the risks of developing PM and DM did not differ significantly between AS patients and non-AS individuals [11]. Therefore, more studies are needed to explore whether axSpA may increase the risk of IIM.

In conclusion, the coexistence of IIM and axSpA is very rare. Most of the cases are female, and axSpA generally precedes the occurrence of IIM. Our study provides further evidence that axSpA patients can indeed have IIM. For axSpA patients who have skin rash, ILD, myalgia, or muscle weakness, we should suspect that they have IIM.

## Figures and Tables

**Table 1:** Clinical characteristics of axSpA.

Patients	1	2	3	4	5	6	7
Gender	Male	Female	Male	Female	Female	Female	Male
Onset age of axSpA (years)	22	29	39	32	30	30	16
Low-back pain	Yes	Yes	Yes	Yes	Yes	Yes	Yes
Peripheral arthritis	Yes	No	Yes	No	No	Yes	No
Iritis	Yes	No	Yes	No	Yes	No	No
Family history of axSpA	Yes	Yes	No	Yes	No	No	No
ESR (mm/h)	2	4	15	3	7	42	14
CRP (normal <0.8 mg/L)	0.258	0.622	1.33	0.151	0.241	0.274	1.84
HLA-B27	Yes	Yes	Yes	Yes	No	No	No
CT/MRI	Grade 4	Grade 3	Grade 2	Grade 3	Grade 2	Grade 2	Grade 3
BASDAI at the IIM onset	3.8	3.4	3.9	1.5	2.8	4.8	0.6
Treatment	THD	IFX	Etanercept, SSZ	NSAIDs	NSAIDs	NSAIDs	NSAIDs

BASDAI: Bath Ankylosing Spondylitis Disease Activity Index; THD: thalidomide; IFX: infliximab; SSZ: sulfasalazine; NSAIDs: nonsteroidal anti-inflammatory drugs.

**Table 2:** Clinical symptoms, findings, laboratory test results, and treatment of IIM patients.

Patients	1	2	3	4	5	6	7
Gender	Male	Female	Male	Female	Female	Female	Male
Onset age of IIM	28	49	55	32	44	30	26
Skin rash	Gottron sign	No	Gottron sign	No	No	Heliotrope sign	No
Muscle weakness	Grade 3	Grade 3	Grade 5	Grade 4	Grade 3	Grade 4	Grade 5
MSA	No	SRP	MDA5	No	No	MDA5	PL-7
EMG	MD	MD	NA	MD	MD	NA	MD
Muscle MRI	NA	IE	NA	NA	Normal	IE	IE
Muscle biopsy	PA	Necrosis	PA	Necrosis	Necrosis	Inflammatory infiltration	PA
ILD	No	NSIP	OP	No	No	OP	No
CK (U/L)	87	1125	59	1352	2998	32	481
ALT (U/L)	20	248	85	98	27	98	27
AST (U/L)	17	126	76	44	41	81	21
LDH(U/L)	169	752	240	631	221	350	208
Treatment	Pred, HCQ	Pred, TAC, TCZ	Pred, CsA	Pred, MTX	Pred, MTX	Pred, CsA, IVIG	Pred
Follow-up time	54 months	36 months	18 months	36 months	36 months	16 months	12 months
Outcome	Improved	Improved	Improved	Improved	Improved	Improved	Improve

CT: computed tomography; MRI: magnetic resonance imaging; IE, inflammatory exudation; NA: not available; ILD: interstitial lung disease; Pred: prednisone; HCQ: hydroxychloroquine; MTX: methotrexate; CsA: cyclosporine; TAC: tacrolimus; TCZ: tocilizumab; MSA: muscle special antibody; PA: perifascicular atrophy; EMG: electromyography; MD, myogenic damage; NSIP, nonspecific interstitial pneumonia; OP, organizing pneumonia.

## References

[1] Prohaska E., Moritz E. (1983). Combination of ankylosing spondylitis with dermatomyositis and atypical gout (isolated gout kidney). *Zeitschrift für Rheumatologie*.

[2] Chadrasekhara P., Jayachandran N., Rajasekharan L., Narsimulu G. (2006). P71 SLE, dermatomyositis and ankylosing spondylitis overlap-a case report. *Indian Journal of Rheumatology*.

[3] Sattar M. A., Al-Sughyer A. A., Siboo R. (1988). Coexistence of rheumatoid arthritis, ankylosing spondylitis and dermatomyositis in a patient with diabetes mellitus and the associated linked HLA antigens. *Rheumatology*.

[4] Rudwaleit M., Landewé R., Van Der Heijde D. (2009). The development of assessment of spondyloarthritis international society classification criteria for axial spondyloarthritis (part I): classification of paper patients by expert opinion including uncertainty appraisal. *Annals of the Rheumatic Diseases*.

[5] Rudwaleit M., Van Der Heijde D., Landewé R. (2009). The development of assessment of spondyloarthritis international society classification criteria for axial spondyloarthritis (part II): validation and final selection. *Annals of the Rheumatic Diseases*.

[6] Bohan A., Peter J. B. (1975). Polymyositis and dermatomyositis. *New England Journal of Medicine*.

[7] Bohan A., Peter J. B. (1975). Polymyositis and dermatomyositis (second of two parts). *The New England Journal of Medicine*.

[8] Hoogendijk J. E., Amato A. A., Lecky B. R. (2004). 119th ENMC international workshop: trial design in adult idiopathic inflammatory myopathies, with the exception of inclusion body myositis, 10-12 October 2003, Naarden, The Netherlands. *Neuromuscular Disorders*.

[9] Hopkins G. O., McDougall J., Mills K. R., Isenberg D. A., Ebringer A. (1983). Muscle changes in ankylosing spondylitis. *Rheumatology*.

[10] Faus-Riera S., Martínez-Pardo S., Blanch-Rubió J. (1991). Muscle pathology in ankylosing spondylitis: clinical, enzymatic, electromyographic and histologic correlation. *The Journal of Rheumatology*.

[11] Chen H.-H., Chao W.-C., Chen Y.-H. (2019). Risk of immune-mediated inflammatory diseases in newly diagnosed ankylosing spondylitis patients: a population-based matched cohort study. *Arthritis Research & Therapy*.

